# Climate science curricula in Canadian secondary schools focus on human warming, not scientific consensus, impacts or solutions

**DOI:** 10.1371/journal.pone.0218305

**Published:** 2019-07-18

**Authors:** Seth Wynes, Kimberly A. Nicholas

**Affiliations:** Lund University, Centre for Sustainability Studies, Lund, Sweden; Royal Holloway University of London, UNITED KINGDOM

## Abstract

Despite an overwhelming scientific consensus that climate change poses severe risks to human and natural systems, many young Canadian adults do not view it as a major issue. We analyzed secondary science curricula in each province for their coverage of climate change according to six core topics: physical climate mechanisms (“It’s climate”), observed increase in temperature (“It’s warming”), anthropogenic causes of warming (“It’s us”), scientific consensus (“Experts agree”), negative consequences associated with warming (“It’s bad”), and the possibility for avoiding the worst effects (“We can fix it”). We found that learning objectives tend to focus on knowledge of the first three elements, with little or no emphasis on scientific consensus, climate change impacts, or ways to address the issue. The provinces of Saskatchewan and Ontario provide the most comprehensive standards for climate change education, while Nova Scotia and New Brunswick provide the least. We conducted interviews with individuals responsible for curriculum design in six different provinces to understand how curriculum documents are developed and whether political controversies influence the writing process. Interviewees described a process relying on input from professionals, institutions, and members of the public where curriculum developers made decisions independent of political concerns. In some cases, efforts to provide balance may have led to a focus on social controversy, contrary to overwhelming scientific consensus. Curriculum documents are the basis for teacher instruction and textbook content; aligning these documents with the best possible evidence can improve student learning and engage the next generation of Canadians on the critical issue of climate change.

## Introduction

A host of political issues demanding some level of scientific literacy face the present and future citizens of any democratic country. We take scientific literacy to mean “a civic competency required for rational thinking about science in relation to personal, social, political, economic problems, and issues that one is likely to meet throughout life” [1]. Climate change is an example of a global issue with ramifications in the personal, social, political and economic spheres of life.

Planetary warming brought on by human emissions of greenhouse gases (GHGs) has begun and as temperatures increase, so do the risks of coastal flooding, food insecurity, and deaths from extreme heat [2]. Meeting the Paris temperature targets will require at least 50% reduction every decade in gross CO_2_ emissions [3], a task that will entail behavioural changes from citizens (especially those in developed countries like Canada) [4, 5]. At present, the Canadian government is attempting to reduce its greenhouse gas emissions through a price on carbon [6], while simultaneously expanding its fossil fuel infrastructure [7, 8], in contradiction to its climate goals. In the future, governments and citizens may be asked to weigh the merit of high-risk geoengineering schemes that offer a final chance to act on climate change if mitigation efforts across society have proved insufficient to meet internationally agreed climate targets [9, 10]. Since these critical issues will persist for decades, climate change education will continue to be necessary for Canadian citizens for the foreseeable future.

Through its rhetoric and its international commitments, the government of Canada has shown that it regards the issue of climate change as a serious, global threat. The official website of the government agency, Environment Canada says “The scientific evidence is clear: climate change is one of the greatest threats of our time” [11]. In his speech at the United Nations Paris Climate Summit (COP 21), Prime Minister Justin Trudeau said, “Canada can and will do more to address the global challenge of climate change. We will do so because the science is indisputable, and tells us that our planet is already changing in ways that will have profound impacts on our future… Our government is making climate change a top priority” [12]. The Canadian Government has also supported an ambitious target to limit global warming to 1.5°C [13].

Yet there appears to be a gap between what the government says and what its citizens believe. Only 61% of Canadians believe that “the science is conclusive that global warming is happening and caused mostly by human activity” [14]. That figure increases to 78% when considering the age group 18–24, but 15% of those young adults believe that climate change may not be caused by humans, and 7% aren’t sure that climate change is happening at all [14]. That same demographic scores low on concern for climate change, with 52% falling in the range between “somewhat concerned” and “not at all concerned” about climate change [14]. Not long ago the vast majority of those young people were school students, and should have received education on climate change as part of compulsory education, as evidenced by Canada’s low drop-out rates [15]. It stands to reason that education in Canada has not adequately convinced young people of the scope and urgency of this issue.

Unlike the Canadian public, the scientific community shows strong agreement on human-caused climate change and its increasingly harmful effects. An analysis of 928 peer-reviewed journal articles on the subject of climate change found that none explicitly rejected the consensus view that humans were causing climate change [16]. A more recent study of 11 944 peer-reviewed climate science abstracts found that of those papers which expressed a view on anthropogenic climate change, 97% supported the view that humans are causing climate change [17]. Others found that the level of agreement on the subject of anthropogenic climate change increases with the level of expertise on the subject [18, 19]. The evidence therefore shows a scientific consensus (which we define as general agreement amongst scientists as expressed in peer-reviewed literature) that humans are causing climate change. Further, comprehensive scientific assessments from the Intergovernmental Panel on Climate Change (IPCC) warn of increasing “the likelihood of severe, pervasive, and irreversible impacts for people and ecosystems” [20] from continued warming. Human impacts from climate change have already been observed, but risks to health, livelihood, water security, and economic growth are projected to increase with greater warming, with high or very high risks across sectors and regions illustrated by the “Reasons for Concern” at 2°C of warming [21].

The context of the problem can therefore be summarized as this: climate change poses huge risks to humanity and the biosphere which are recognized both by scientists and the Canadian government, and yet survey data show that even those Canadians who have received education on climate change often do not support this scientific consensus. As public opinion and collective action are important precursors to policy change [22], raising Canadians’ environmental citizenship (“those values and practices appropriate to the achievement of sustainability” [23]) may increase the motivation of government officials to act to mitigate emissions in line with their pledged reductions.

In Canada, fostering responsible environmental citizenship by students on issues such as climate change is a goal stated in several important government documents. For instance, the Common Framework of Science Learning Outcomes K-12, a foundational document for curriculum design in Canada, says, “Students will be encouraged to develop attitudes that support the responsible acquisition and application of scientific and technological knowledge to the mutual benefit of self, society, and the environment” [24]. Some provinces provide more detail; the Ontario curriculum guidance document “Shaping Our Schools, Shaping Our Future” says that environmental education “seeks to promote an appreciation and understanding of, and concern for, the environment, and to foster informed, engaged, and responsible environmental citizenship” [25].

In contrast with Canada’s ambitious goals for fostering environmental citizenship, research suggests this topic is often added to curricula as an afterthought. For example, an analysis of Turkish and Bulgarian curricula found that substantially more attention is paid to knowledge than environmentally responsible behaviors [26]. Researchers analyzing climate change in the science curriculum in Singapore described learning objectives that were focused on content knowledge ranging from the carbon cycle to the human impact on climate change, with no mention of how students could positively participate in this issue [27]. A study of the national curriculum in England and Wales found that those guidelines that teachers are legally bound to teach focus on information, whereas topics like citizenship or education for sustainable development are suggested only if time and space allows [28]. Our previous work on Canadian science textbooks found that they focus on low or medium-impact individual climate actions such as recycling or changing lightbulbs, and only infrequently mention the behaviours that most reduce a person’s carbon footprint, such as living car, flight, and meat-free [5].

To investigate how well existing education aligns with international scientific consensus and national policy goals in Canada, in this paper we analyze how secondary school climate change curricula are designed, noting areas where educational guidelines are firmly supported by scientific evidence and where they are not. We also make recommendations for changes that could be made without the need for greater alterations in the educational system. Our research questions are as follows:

1. What climate change topics are covered in different Canadian secondary curriculum materials?
2. How are Canadian climate change curricula developed?
3. How do textbooks and curriculum documents address controversy about climate change?

## Methods

### Case description

Five million students are enrolled in public schools in Canada, with approximately 350 000 students graduating from secondary school in a typical year [29]. Canada is divided into ten provinces and three territories, each of which controls its own educational system. (As the territories are sparsely populated and mostly borrow educational materials from nearby provinces, in this paper we sometimes reference only “provinces” for the sake of simplicity). Education can vary between the provinces, resulting in different curriculum documents, standardized tests, teacher education programs and so forth. Within these educational systems, science is split into ‘silo’ subjects; chemistry, biology and physics are common, while earth and space science or environmental science are less likely to be present at all grade levels. Teachers at the secondary level are subject specialists, meaning they are trained to teach a few specific subjects, rather than all subjects. Amongst science teachers, for example, some are qualified to teach chemistry, but not biology or physics. This is important for climate change, which is not a ‘silo’ subject, but is instead interdisciplinary in nature.

### Curriculum analysis

In order to analyze the high school science curricula from the 13 Canadian provinces and territories, we grouped learning objectives into six core topics (see Table 1) associated with statements from the IPCC Fifth Assessment report [30]. The IPCC makes use of a rigorous peer review process featuring 600 authors and several rounds of review, which is why it is regarded as the most reputable source of scientific understanding on climate change [31]. Understanding of five of the six core topics has also been associated with support for climate change mitigation policies (see Table 1). The topics include: foundational knowledge of the physical climate system (abbreviated as “It’s climate”), observations and proxies of rising temperature (“It’s warming”), anthropogenic contributions to climate change (“It’s us”), the scientific process of obtaining consensus (“Experts agree”), the various impacts associated with climate change (“It’s bad”), and the approaches and policies that can be used for mitigation (“We can fix it”).

**Table 1:** Framework for evaluating the scope of climate change knowledge addressed by secondary school curriculum documents. The first topic was added based on identifying widespread coverage in teaching materials after [30]. The remaining five topics are summarized from research on public attitude and policy preferences [34], with wording modified by comments on social media [35].

Core Topic	Evidence	Evidence for effectiveness of this message in behaviour/attitude change	Example of relevant learning objective
It’s climate	Well-established physical principles, e.g., heat-trapping effects of greenhouse gases	N/A	Outline factors influencing the Earth’s radiation budget. (Manitoba Gr. 10 Science, S2-4-02)
It’s warming	Globally averaged land and ocean temperature increased by 0.85°C from 1880 to 2012, based on multiple, independent datasets [36]. More recent estimates are for approximately 1.0°C of global warming above pre-industrial levels [21].	[34] [37] [38]	Conduct an inquiry to determine how different factors (e.g., an increase in surface temperature, an increase in water temperature) affect global warming and climate change (Ontario Gr. 10 Science, D2.4)
It’s us	Humans are the main cause of current warming, 95% certainty [36]	[34] [37] [38]	Provide examples of human actions that have contributed to the anthropogenic greenhouse effect. (Saskatchewan Gr. 10 Science, SCI10-CD1g)
Experts agree	Zero out of 928 peer reviewed studies denied human role in climate change [16] Degree of certainty in human role in climate change increases with increasing expertise [18] 97% of 11944 peer-reviewed abstracts support human role in climate change [17]	[39] [40] [37]	Examine the role of policies, summits, models, and organizations … in obtaining a high degree of consensus among scientists regarding anthropogenic climate change. (Saskatchewan Environmental Science 20, ES20-AS2c)
It’s bad	Cost of 5–10% of annual global GDP if climate change unchecked [41] Extreme heat events will become more frequent and global sea level will continue to rise [36] ”extinction risks will accelerate with future global temperatures, threatening up to one in six species” [42]	[34] [38]	Describe the impacts of climate change in Canada on human health. Include: (i) heat stress (ii) migration of diseases (Newfoundland and Labrador Environmental Science 3205, 5.24)
We can fix it	“CO_2_ emissions reductions that limit global warming to 1.5°C with no or limited overshoot can involve different portfolios of mitigation measures, striking different balances between lowering energy and resource intensity, rate of decarbonization, and the reliance on carbon dioxide removal” [21] Barriers to 100% conversion to wind, water and solar energy are mostly political and social not technical [43]	[34] [38] [44]	Assess, on the basis of research, the effectiveness of some current individual, regional, national, or international initiatives that address the issue of climate change (Ontario Gr. 10 Science, D1.2)

To summarize the current scientific consensus on climate change: warming of the climate is unequivocal [31] and its human cause has been established by exhaustive analyses of scientific literature and the scientific community [32]. While some regions and sectors may experience slight benefits from climate change, a review of 3280 research papers concluded the overwhelming impacts on human and natural systems are negative and that the “small set of positive and neutral impacts cannot counterbalance any of the many detrimental impacts that were uncovered in our literature search, particularly when many of these impacts are related to the loss of human lives, basic supplies such as food and water, and undesired states for human welfare such as access to jobs, revenue and security” [33]. In terms of human health for instance, the IPCC projects that “Any increase in global warming is projected to affect human health, with primarily negative consequences” [21]. Looking ahead, staying below the internationally agreed temperature targets will reduce risks and requires deep cuts in greenhouse gas emissions within the coming decade [21].

To determine the extent to which different provinces were covering the current state of climate change knowledge (Column 1 of Table 1), we read all high school science curriculum documents, and divided their relevant learning objectives into the six core topics. Learning objectives were not included if climate change was referenced as only one of several possible examples. For instance, in the Gr. 12 University/College Science Preparation course (SNC4M), one learning objective says, “explain the impact of various threats to public health, including infectious diseases (e.g., hepatitis, HIV/AIDS, tuberculosis, malaria, sexually transmitted diseases), chronic diseases (e.g., cardiovascular disease, diabetes, asthma), and environmental factors (e.g., climate change, air pollution, chemical pollutants, radiation)” [45]. Although climate change is listed as a threat to public health (a statement that could fall under “It’s Bad”), in this case, because a teacher could choose many other examples to meet the objective instead, it did not count in our assessment. Similarly, if climate change was mentioned in supporting documentation (suggested resources, optional activities etc.), it was not counted towards any of the categories. Lastly, we noted when a course was mandatory for graduation from secondary school, or merely optional.

Canada has a parliamentary democratic system, with five major political parties in government as of early 2019. The parties range from the Conservative Party of Canada (CPC), whose leadership candidates mostly argue against climate change mitigation measures [46], to the Green Party of Canada who propose halting expansion of the country’s lucrative oil sands [47]. More generally, the strength of Green Parties in democratic systems has been found to have a positive effect on measurable environmental indicators such as air quality [48] while political conservativism has been associated with lower levels of environmental concern [49, 50] and lower levels of concern for climate change, even across different nations [14, 51]. We therefore compared the provincial coverage of climate change in the curricula documents with the per capita greenhouse gas (GHG) emissions of the provinces, as well as with the breakdown of votes in the last federal election to search for indications that political forces influenced curriculum design. In undertaking this analysis, we suspected that certain provinces with a history of fossil fuel extraction (e.g. Alberta, Saskatchewan) would have less coverage of climate change in their curricula than provinces that have enacted climate policies such as a carbon tax (e.g. British Columbia, Ontario).

### Curriculum development

To gain a better understanding of the cause of differences between the provincial curricula, we asked contributors to each of the province’s science curriculum to participate in interviews. In total, representatives from six of the ten provinces agreed to interview. Interviewees included both contributing teachers as well as science consultants. Although the study scope exempted it from Swedish requirements for formal ethical review by an institutional review board, all procedures performed in this study involving human participants were in accordance with the ethical standards of the institution, and with the 1964 Helsinki declaration and its later amendments or comparable ethical standards. Prior, written informed consent was obtained from all individual participants included in the study (See consent form in S1 Text).

The interviews were semi-structured, with questions provided beforehand to encourage thoughtful responses as some questions were difficult or referred to events that took place several years in the past (see S2 Text for interview questions). One potential explanation for provinces having dissimilar approaches to climate change could be that political differences between provinces result in different outcomes. For instance, in the United States, controversial issues such as evolution often result in school board policies or statewide educational legislation that causes education to differ greatly between jurisdictions [52]. Additionally, the curriculum could be politically influenced without legislative force; internal political pressure from high-ranking public officials, NGOs, or concerned parents or members of the public might also result in more or less coverage of climate change (for instance). Interviewees were therefore asked if any political controversies around climate change affected their curriculum writing process. Further questions explored other possibilities for differences between provincial coverage of climate change, such as the use of government documents, the way in which curriculum writers are selected, the time period in which the curriculum was written, and the overall curriculum design process.

The interviews lasted approximately thirty minutes and were conducted via telephone or Skype depending on the preference of the interviewee, with one interviewee choosing to respond in written form. Interviews were recorded and then transcribed with pseudonyms and with identifying information removed. Transcripts of the interviews were provided to the interviewees. Those parts which would be used as quotations in the final publication were indicated separately for interviewees who then approved the accuracy of each quotation. Thematic analysis was used to analyze the interview transcripts, according to guidelines provided by Bryman [53]. Following transcription of the recordings we tentatively identified common themes from the interviews. Some themes were simply straightforward responses to questions (such as whether controversy on the subject of climate change affected the curriculum design), while others were unsolicited ideas such as the need to teach about sustainability. We arranged the themes as rows in a spreadsheet and pasted relevant interviewee quotations into adjacent columns. While this type of qualitative analysis with a small sample does not definitively indicate trends, nonetheless we report when many of the interviewees responded in similar ways to the same question or mentioned similar themes without prompting, as an indication of which trends or practices were more salient in the curriculum design process.

### Controversies in textbooks and curricula

Having assessed the coverage of six topics in research question 1, we then analyzed curricula and textbooks to see how any controversies around climate change were presented. Analysis of curriculum documents for controversy occurred while recording coverage of the six core topics. Textbooks offer greater detail than curriculum documents and therefore can give further indication of the content being communicated to students. Ten textbooks used in seven Canadian provinces were therefore analyzed. Difficulties in obtaining texts used by the provinces of Alberta, Manitoba, and Nova Scotia prevented analysis of texts representing those areas, while the three territories make use of provincial resources that were included in our analysis. Those chapters pertaining to climate change were read in their entirety, and statements which encouraged debate or controversy on the subject of climate change were noted, with examples described below.

## Results

### Curriculum analysis

**Fig 1:**
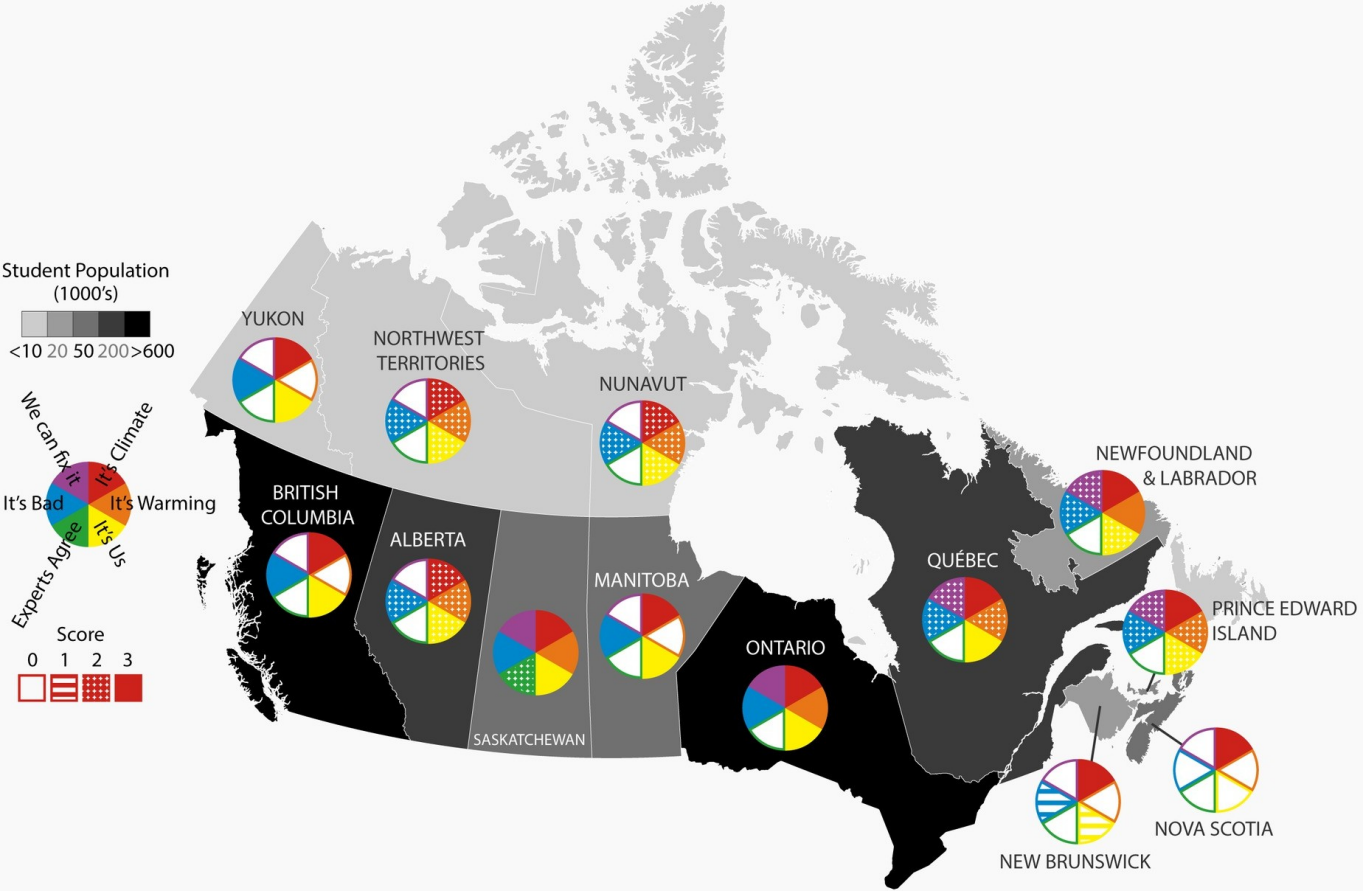
Map showing provincial coverage of climate change in curriculum documents as of 2018. Solid colours (score of 3) indicate that a core teaching topic is covered in a mandatory course in the curriculum. A score of 2 (light hashing) indicates coverage in a non-mandatory course, a score of 1 (lines) indicates coverage in an optional unit, and a score of 0 indicates no coverage. Note that there are large disparities in the number of inhabitants of different regions (student populations are available in Table 2). A new curriculum is planned for British Columbia starting 2019/2020 school year, see Discussion. Figure by Emma Li Johansson.

In analyzing the content of Canadian science curricula against six core topics, every province covered “It’s climate,” while the categories “We can fix it” (five out of 13 provinces) and “Experts agree” (only one province, Saskatchewan) were least represented (Fig 1). The greenhouse effect was the most commonly addressed topic amongst different provinces, with every province and territory covering the subject, and most in a mandatory course. Three provinces (Alberta, Northwest Territories and Yukon) only covered climate change in non-mandatory courses. Saskatchewan and Ontario had the most comprehensive coverage of climate change with five of the six categories covered in mandatory courses. New Brunswick and Nova Scotia had the least comprehensive coverage with only “It’s climate” addressed in mandatory secondary science courses.

We expected to find that those provinces that were more politically conservative or had a larger fossil fuel industry would have less climate change coverage. Yet Saskatchewan, which has the highest per capita GHG emissions (due to the presence of fossil fuel extractive industries) and the second highest support for the Conservative Party of Canada, had the most comprehensive coverage of climate change in curriculum documents (Table 2). Alberta is similarly positioned in terms of GHG emissions and political conservativism but has roughly the same curricular coverage of climate change as British Columbia (which has the highest proportion of Green Party voters in the most recent federal election).

**Table 2:** A comparison of key data for the ten Canadian provinces and three territories.

Province	Number of secondary students (grades 9–12 in 2016/17)^a^ [54]	Coverage of climate change curriculum categories^b^ (Fig 1)	Publication date of main curriculum document	GHG emissions per capita (tonnes) [55]	Number of seats above a majority for major conservative party in province^c^	Percent vote by party, 2015 Federal Election^d^ [56]				
						Green Party	NDP	Bloc Québécois	Liberal Party	CPC
Saskatchewan	53,193	6	2014	67.3	19	2.1	25.1	-	23.9	48.5
Ontario	629,148	5	2008	12.4	-28	2.8	16.6	-	44.8	35.1
Newfoundland and Labrador	21,141	5	2010	20.0	9	1.1	21.1	-	64.5	10.3
Quebec	180,048	5	2014	10.1	-54	2.8	25.4	19.4	35.7	16.7
Prince Edward Island	6,150	5	2011	12.3	-10	6.0	16.0	-	58.3	19.3
Alberta	202,659	4	2005*	66.4	32	2.5	11.6	-	24.5	59.6
Northwest Territories	2,856	4	2005	34.1	32	2.8	30.5	-	48.3	18.3
Nunavut	3,123	4	2005	8.3	32	1.5	26.6	-	47.1	24.8
British Columbia	185,505	3	2008**	13.6	6	8.2	26.0	-	35.1	29.9
Manitoba	59,571	3	2001	16.8	-5	3.2	13.6	-	44.7	37.4
Yukon	1,566	3	2008	8.1	0	2.8	19.4	-	53.7	24.3
New Brunswick	32,214	1	2002	19.7	16	4.7	18.4	-	51.6	25.4
Nova Scotia	38,328	1	2012	17.6	-17	3.4	16.3	-	62.0	17.9

^a^Note that secondary school in Quebec only goes to Grade 11

^b^Coverage indicates the number of climate change curriculum categories addressed in mandatory or non-mandatory courses

^c^In election preceding creation of curriculum document. Nunavut and NWT have non-partisan legislatures and were assigned the same value as Alberta (whose curriculum documents they borrow)

^d^Political parties are ordered roughly from political left to right. Voting information retrieved from Elections Canada

* Updated in 2014, but changes were not related to relevant content.

**Update planned for 2019/2020 school year, see Discussion

We conclude that there is no relationship between either per capita GHG emissions or political conservativism and climate change coverage in curriculum documents in Canada. Instead, there may be a weak relationship between the time that curriculum documents were written and how comprehensively they address climate change. The documents with the two oldest curriculum documents (Manitoba and New Brunswick) have among the lowest coverage of climate change. While our sample size is small, we nevertheless tested for correlations between curriculum coverage (column 3 of Table 2) and publication date of the document as well as per capita GHG emissions in each province, finding no significant relationships. We also tested for a correlation between the margin of victory (or defeat) of the major conservative party (CPC) in the election preceding the creation of each curriculum document and curriculum coverage, also finding no significant correlation (r = -0.07, p = 0.81). (The most recent election results are shown in Table 2).

### Curriculum development

Three male and three female interviewees, from six different provinces, with a range of 8 to 35 years of experience as educators, participated in the interviews. Interviews revealed moderate differences in the curriculum development process that appear to be responsible for the variations between provincial curriculum documents on the topic of climate change (as opposed to external factors such as political input).

According to interviewees, the process for selecting individuals to contribute to curriculum design varied from several different formal application processes to recommendations from school boards to hiring due to circumstance. This could introduce an element of randomness that would partially explain some interprovincial disparities, especially as one interviewee commented that “…it’s really the luck of the draw who ends up on a committee and what they’re interested in can absolutely, totally change the direction of what a course might be.” Although there is a larger framework, called the Common Framework of Science Learning Outcomes K to 12, which provides guidance [24], some provinces rely on it more than others, and one interviewee noted that it provides little detail beyond grade 10.

Interviewees reported that numerous factors influence how much space in the curriculum is given to climate change including: feedback from teachers; input from university faculties of education, researchers and environmental groups; the approaches taken by other provinces and jurisdictions; and the availability of teaching resources (textbooks, etc.) for the subject. While interviewees acknowledged that climate change can be politically controversial, and that pressures regarding curriculum content are applied from government officials, NGOs and the public, no interviewee described direct interference in the curriculum writing process (Box 1).

**Box 1. Interviewees on the topic of addressing external pressures when designing curriculum documents, responding to the question from the researcher: “Politically, climate change is a very controversial topic. Did that influence the approach taken to writing parts of the curriculum that related to climate change?” (if so: “How?”)**

Interviewee 4: …politics doesn’t influence when it comes to that aspect of it. That aspect of writing curriculum.

Interviewee 1: We weren’t being told by the government—we had a lot of freedom with the document.

Interviewee 2: There wasn’t really a place to go that said, “This is a good thing to do”. There certainly wasn’t any pressure at any government level to say, “You must incorporate climate change in your curriculum”.

Interviewee 2: So we do get questions asked a lot from the Ministry of the Environment, but no direction.

Interviewee 3: It can weigh into our considerations if we have groups come forward and exert pressure to include certain areas in the curriculum. But we really look for a balanced approach to the curriculum.

Interviewee 6: …but nobody tells us that you have to teach this. But on the other hand there are so many, many groups that want you to teach what they want. Like…

Interviewer: NGOs?

Interviewee 6: Environmental groups, yeah, NGOs. So many of all kinds, that want you to teach whatever, that don’t understand why you’re not doing X, Y or Z. Not just for science, for everything. But certainly for all the sciences also.

Interviewee 5: No, climate change was included as a factual component of the curriculum based on the recommendation from [name of provincial report].

Instead, five of the six interviewees made comments about how documents in other jurisdictions and provinces can influence the curriculum writing process. Often, a scan of educational materials from other locations was one of the first steps mentioned in the development process. As an example of this interprovincial relationship, the science consultant for one province described a conversation with colleagues from another province about a change in senior science curriculum to emphasize courses based on subjects or problems rather than traditional subjects like chemistry: “every single one of them said, ‘Well, we’d never pull this off in our province politically, but if you can get it going in [your province], then we might be able to afterwards.’” Overall, we found that in Canadian climate change curricula, external influence is minimal, but change in one province may give momentum to other provinces considering change in the same direction.

Another recurring theme amongst curriculum developers was the belief that educators had a responsibility to dispassionately provide students with information, or that students needed to learn the right skills to evaluate the issue of climate change for themselves (Box 2).

**Box 2. Curriculum developers on a balanced approach to climate change education**

Interviewee 1: So is it, is it a political issue? Yeah, absolutely. How you deal with it–it has to be a very balanced approach.

Interviewee 1: Like I said, you basically have to be fence-sitting even if you’re very passionate about these issues. And I know that it’s probably not that easy with some teachers, and it’s probably not even always easy with me on some issues because I get so passionate about them. I, you know, I weigh in on it. But at the end of the day it has to be a very neutral approach otherwise you’re going to introduce a bias into all of these documents that you’ve put together.

Interviewee 2: I think we’ve sort of minimized the concern out there, and I’m sure there’s some groups would like a little more on climate change. But again, I’ll say to them, “Our job’s to open the door to talk about it, … our job as teachers isn’t to say climate change is good or bad, right?” It’s to say, “We want students to understand how scientists develop the information that they do.”

Interviewee 3: …so I think that, yes, the fact that people can have strong interests and can try to sway individuals for favour, for power, for money or whatever—we want students to be able to have the skills to be able to sift through information and make informed decisions that will enable them to survive better and flourish.

Interviewee 4: I always tell my students, I may share my opinion, but it’s nothing more than, and I never ask them to agree with me. In fact, I would prefer for them—my goal is to say, “this is just one more piece of information that you have got to verify and agree or disagree with.”

### Controversies in curricula and textbooks

#### Controversies in curricula

Manitoba’s science curriculum, which is among the oldest in Canada (dating from 2001), displayed a tendency to depart from the scientific consensus. For instance, specific learning objectives moderated the idea of anthropogenic climate change, saying that the climate “can be influenced” by humans [57] rather than describing humans as the major driver of current climate change. But divergence from scientific understanding was much more pronounced in supplementary materials in Manitoba’s curriculum, which recommended reading material produced by “Friends of Science”, an organization described as opposing the IPCC understanding of climate change [58]. The curriculum document adds, “It should be noted that there is significantly polarized debate on the issue among scientists. Students should be justifiably cautious about accepting unsubstantiated claims about global warming” [58]. This is particularly concerning given that there actually is no polarized debate in the scientific community [17]. Furthermore, individuals who hold the misconception that there is widespread disagreement amongst scientists on this issue are less likely to support policies to mitigate climate change [39].

Manitoba was not the only province with curriculum documents conveying disagreement amongst the scientific community (Table 3). The supporting documentation for both Newfoundland and Prince Edward Island undermine the notion of a scientific consensus, suggesting that debate should be encouraged amongst students on the cause of current climate change. This raises the question of how climate change is best taught, and whether debate on the biophysical existence and anthropogenic cause of warming should encouraged on the issue, as it may lead students to incorrectly conclude that there remains scientific disagreement on these topics.

**Table 3:** Statements in curriculum documents that oppose the scientific consensus on anthropogenic climate change.

**Manitoba**	“It is important for students to conduct research that is fair and representative of alternative, and viable, scientific viewpoints on such a vital issue. Students should research climate science, as articulated by organizations such as The Friends of Science—Providing Insight into Climate Science (see <http://www.friendsofscience.org/>). This large, international community of climate scientists, for instance, holds views quite contrary to what has been supported by Environment Canada and the United Nations’ (UN) Intergovernmental Panel on Climate Change (IPCC) over the past decade … Students could role-play disparate points of view within the climate science community.” [58] “A discussion of the merits and shortcomings of the scientific community’s research agenda into the anthropogenic CO_2_ contribution to potential global warming could be conducted in relation to this topic. It should be noted that there is significantly polarized debate on the issue among scientists. Students should be justifiably cautious about accepting unsubstantiated claims about global warming. This issue provides an opportunity to engage students in the patterns of behaviour that occur within science during what can be termed a “crisis” situation.” [58]
**Newfoundland and Labrador + Prince Edward Island**	“Teachers should ensure that students understand that there are contradictory viewpoints to this issue. Students may have seen Al Gore’s movie “An Inconvenient Trust [*sic*]” which explains the anthropogenic side of the story. Teachers should ensure that students know that there may be a natural cyclic event on Earth causing global warming. Some sources include volcanic activity, ocean currents, solar variability, Earth’s orbit and tilt, plate tectonics and biological evolution.” [59] “Not all scientists agree on the science surrounding climate change. Some scientists are skeptical and believe that climate change is a natural, cyclic process. Research and try to find opposing arguments to climate change. Take one side of the issue and debate it with another student with an opposing viewpoint.” [59] and [60]

While some provinces (and textbooks) suggest teaching the existence of human-caused climate warming (“It’s Us”) as a debate, few are teaching the scientific consensus of the current human-caused climate warming. Only one province (Saskatchewan) covered the core teaching topic “Experts agree”, and only in a non-mandatory course (Fig 1). The “We can fix it” topic was also sparsely covered, with only five provinces addressing this concept in their objectives. But the way in which “We can fix it” is covered is also an area of interest. For instance, some provincial curricula suggest individual actions that would lead to climate change mitigation [61], largely focusing on energy efficiency and recycling, while others discuss the political steps that can be taken [59]. But no province connects these two things–i.e., how can an individual contribute to political or structural changes. Some textbooks fill these gaps, discussing both the meaning [62] and existence of scientific consensus [63], as well as prompting students to participate in political processes: “Make a list of five ways you can reduce your personal impact on climate change and three ways you can influence corporate and/or governmental action of climate change” [64]. So even with little prompting from the curriculum, some textbooks encourage positive personal and political participation regarding climate change.

#### Controversies in textbooks

Textbooks are created as a resource available to teachers based on the learning objectives provided in curriculum documents. In our textbook analysis we noted statements that suggest debate or controversy on subjects that the majority of scientists would agree upon (Table 4). In these examples, doubt is cast on the consensus position that humans are driving climate change (“It’s us”) as well as on the scientific consensus itself (“Experts agree”). Benefits from climate change are discussed, without a clear context to show that they are far outweighed by costs. The three textbooks cited were written for the Ontario curriculum.

**Table 4:** Statements in Canadian science textbooks suggesting controversy on climate change unsupported by scientific consensus.

Core topic	Textbook quotation	Citation
It’s us	Human activities, such as the burning of fossil fuels, releases [*sic*] carbon dioxide into the atmosphere, which may result in climate change.	[65]
	Scientists are still debating whether climate change is affected more by slow, gradual changes or by sudden, catastrophic changes. This question is at the centre of the controversy about whether humans can cause Earth’s climate to change significantly.	[65]
	However, some people argue that today’s global warming could just be part of a natural climate cycle that occurs over thousands of years. They believe that until such cycles are fully described, the human contribution to global warming remains debatable…	[64]
	Climate skeptics … make three main points. • We do not understand Earth’s climate well enough to make predictions about the future. • The global climate is getting warmer but not because of human activities. • The global climate is getting warmer, but this will create greater benefits than costs.	[64]
Experts Agree	“Scientists Disagree Over Global Warming.” “Future Climate Uncertain.” You may have seen headlines like these on web sites or in newspapers or magazines, or heard similar claims in the media. Both statements are true. However, non-scientists and scientists often interpret disagreements and uncertainties in different ways.	[65]
It’s Bad	Most discussions of climate change give the impression that the impacts of climate change will always be negative. However, there may also be some positive impacts.	[63]
	Possible benefits of Climate Change in the Arctic … Less sea ice means that it will become easier for ships to reach the Arctic and the valuable resources there. In addition, ships could follow much shorter routes by travelling across the Arctic through the Northwest Passage rather than taking longer, more southern routes.	[63]
	Not all the projected effects of climate change are negative. Ontario is a major farming province, with over 82 000 farmers and 5.5 million hectares under cultivation … As climate change brings warmer temperatures, the length of the growing season will increase, and farmers will be able to increase crop yields and grow crops that require more heat. As the sea ice on the Arctic Ocean melts, the Northwest Passage shipping route will be open water every summer. Sailing through the Arctic islands will substantially shorten the shipping distance from Europe to China and Japan, reducing the cost of transporting goods. Cruise ships can sail farther north than before, so tourists can follow in the wake of Arctic explorers such as Henry Hudson and John Franklin.	[64]

## Discussion

We found that most Canadian secondary school curricula did not provide full coverage of six core topics associated with increased concern for the issue of climate change. Our analysis of curriculum documents revealed no clear pattern between the politics of a province and their curricular coverage of climate change. This is consistent with the statements provided by curriculum writers who described a curriculum design process that is independent of partisan direction. Textbooks and curriculum documents, however, often contained statements that might cause students to doubt the very robust existing consensus in the scientific community on the human causes of climate change and its negative impacts and risks.

### Curriculum analysis

Earlier we noted that while scientists and the Canadian government consider climate change to be a serious issue, concern for climate change is not overwhelming amongst young Canadians. One hypothesis that has been considered for the small risk perception associated with climate change is a lack of scientific literacy [66]. Taken further, this hypothesis suggests that in order to solve the issue of apathy towards climate change we need more science education. However, Kahan et al. [67] found that scientific literacy does not correlate with concern for climate change among adults, but instead correlates with high polarization on the issue. However, among children ages 11 to 14, increased knowledge of climate change specifically (not general scientific literacy) is correlated with increased acceptance of the anthropogenic cause of climate change, and risk perception of climate change [68]. It is conjectured that this age-related discrepancy is due to the lack of ideological constraints at a young age. This is further evidenced by examples of teachers who do not themselves believe in anthropogenic climate change delivering climate change material to students who deduce anthropogenic causes independent of the beliefs of their teachers [69]. Since education offers a way to promote acceptance of agreed upon concepts, it is important that teachers are instructed (through curriculum documents) to present the most relevant material regarding climate change.

In our review of curriculum documents, we listed six core teaching topics related to climate change, five of which were associated with support for mitigation policies (see Table 1). The remaining topic (It’s climate) simply describes those learning objectives associated with scientific literacy about the general climate, such as the existence and function of ocean currents, or the greenhouse effect. If scientific knowledge did lead to concern about climate change, then this focus on climate knowledge would be sufficient. However, studies have shown that knowledge about the environment does not lead to action for the environment [70] nor for climate change mitigation [67]. Yet we see that many provinces neglect to choose standards that go beyond scientific literacy and would actually lead to increased concern or action for climate change in their student populations. Instead they focus solely on what Kaiser and Fuhrer [44] describe as declarative knowledge, when procedural or effectiveness knowledge (“We can fix it”) would lead to increased environmental citizenship practices. This is consistent with our review of international studies, which also found an emphasis on knowledge at the expense of civic-oriented teachings. We therefore suggest that as curriculum documents are revised in various provinces, more emphasis should be placed on “It’s warming”, “It’s us”, “It’s bad”, “Experts agree” and especially on “We can fix it”. In this process, the curriculum of Saskatchewan may serve as a model, as it performed best in our analysis.

#### Limitations in the curriculum analysis

This analysis does not provide a complete picture on climate change education in secondary schools in Canada. Only curriculum documents that were available on government websites were examined, which would exclude locally developed courses (courses which are created for a small area or even a single school). International Baccalaureate courses, which are offered at 156 of the 3400 (or 4%) secondary schools in Canada [71], and Advanced Placement courses, which are taken by less than 1% of Canadian secondary students [72], were also not considered. Finally, it should be noted when comparing the different curricula that a province which covers more core teaching topics is not necessarily giving a more thorough treatment of climate change than a province covering fewer topics. For instance, British Columbia covers three topics, but does so in a mandatory course, while Alberta covers four topics, but in a course that not every student will take. In some provinces climate change may have received a more detailed treatment in a different course, for instance in a geography or social science course, or prior to the secondary level. However, we only examined science courses.

Analyses such as this one also have temporal limitations: they are snapshots in time, and curriculum development is an ongoing effort. For instance, in June 2018 British Columbia published a major update to its science curriculum, with a full transition expected by the 2019/2020 school year [73]. The three mandatory learning objectives shown in Fig 1 have been removed from Grade 10 in the draft documents for the new curriculum (though these objectives are retained at the elementary level) while new, optional courses in Grade 11 and 12 cover every core topic except for “Experts agree”. Some of these updates, such as acknowledging the importance of vegetarian diets to climate mitigation, are excellent examples of education making changes to reflect the latest scientific understanding [4, 74, 75]. Because our interviews with curriculum writers referred to the old curriculum documents we make use of the old curriculum in our analysis, but provide details for the new draft documents in S1 Table.

There are also numerous ways of presenting climate change (or environmental education) in the curricula. Some provinces might attempt to infuse such a topic throughout many different courses and grade levels (“mainstreaming”), while others might design one course with a significant environmental component to cover all desired learning objectives. It is also possible that teachers spend more time on environmental issues in other subjects or use climate change as an example in other units in the science curriculum.

But there is some evidence that mainstreaming climate change produces inferior results, and it is better to have dedicated courses or units on important topics. When the Ontario government eliminated the environmental science course, intending for ecological concepts to be taught throughout the curriculum, teachers tended to neglect environmental science in favour of the core curriculum [76]. Another, more recent study consisting of interviews with 11 educators teaching climate change in Alberta found that teachers were already wary of the subject of climate change, partially due to its inherently interdisciplinary nature and the perception of it being a fringe subject in the curriculum [77]. Not all teachers have interdisciplinary training, so spreading environmental issues throughout many courses may result in them not properly addressing those issues. It also eliminates the credibility that the issue gains by being part of the explicit curriculum. Conversely, if teachers receive proper training and feel comfortable integrating these topics, it might prove to be a more effective method. One curriculum development interviewee, for instance, saw the integration of sustainability concepts throughout different grades and courses as a strong, positive characteristic of their province’s education system.

### Curriculum development

In terms of how the curriculum is delivered, and even the practices of those who design the curriculum, Canadian education sometimes tends towards balance rather than evidence (Box 2). Being able to weigh the scientific merit of an argument is certainly a useful skill within scientific literacy, but should students be evaluating balance for issues where a scientific consensus already exists? Some members of society may see climate change as bad while others consider it beneficial, but the scientific evidence overwhelmingly points to negative outcomes both for humanity and the biosphere [33], highlighting that additional warming entails more and longer heatwaves, more intense and more frequent extreme precipitation events, increased ocean acidification and increased sea level rise [20]. With global temperatures threatening to cause extinction for one in six species [42] and 250 000 human deaths per year predicted in the coming decades due to climate change under business as usual emissions [78], an evidence-based approach would need to convey the seriousness of this issue. The desire of curriculum developers to take a balanced, neutral approach to topics that generate political debate is laudable, so long as the balance of evidence presented falls within the boundaries of scientific consensus.

Several curriculum contributors firmly denied feeling any direct political pressure while writing the curriculum. While these interviews were retrospective, and perhaps subject to biases from self-evaluation, the results agree with our curriculum analysis, which shows no obvious relationship between a province’s politics and their approach to climate change. The current curriculum for British Columbia, which was the first province to institute a carbon tax [79] and the only province where a Member of Parliament from the Green Party has been elected [80] has a similar score in our framework as Alberta, which, until 2015, had the same conservative political party in leadership for over forty years [81]. It is reassuring that we found no evidence for the effect of political intervention on curriculum development.

Some curriculum developers noted a conflict between keeping content to a manageable level in courses and including a variety of topics. If climate change is to receive more coverage, especially in provinces which did not address it fully, then curriculum writers would need to be convinced that it truly merits special treatment. But overall, our interviewees report that the curriculum development process seems to function well, encourages a balanced approach, and relies on expert opinion from a variety of sources including researchers and university faculties of education. Combined with the tendency to follow other provinces that are showing leadership on subjects like climate change, we would suggest that this aspect of climate change education will continue to improve as curricula undergo their regular updates and revisions. Perhaps the central flaw with curriculum documents is their turnover time, often more than 15 years (Manitoba and New Brunswick’s curricula were written in 2001 and 2002 respectively). Learning objectives in these documents that run counter to the scientific consensus may be attributed more to age than to intentional misrepresentations. But for a subject like climate change where the science is improving so quickly, updates may be needed more frequently. Additionally, online resources based on the latest peer-reviewed knowledge could be updated with more ease, and professional development could help to bridge the gap between current science and teacher understanding.

### Controversies in curricula and textbooks

Both the textbooks and the curriculum documents we analyzed contained statements that suggest some views outside the scientific consensus on climate change (Table 4), which tend to fall in three categories: (1) disputing the trend (denying that the earth is warming), (2) disputing attribution (denying that warming is caused by humans), or (3) accepting 1 and 2, but believing climate impacts will be delayed or not harmful, and therefore disputing the need for action [82, 83]. Because there have been organized efforts to manufacture uncertainty and controversy by such techniques as cherry-picking studies and manipulating statistical results regarding climate change [84, 85] students may be exposed to arguments that are designed to undermine support for climate science, and the mitigation policies that address the serious consequences of climate change. It is therefore important for climate change communicators and educators to be especially careful of how they address the more controversial aspects of climate change.

While textbooks seemed to encourage controversy, teachers can lead classroom discussion in a way that shows students how the media has heightened controversy, and indeed, teachers may already be doing this. One textbook says, “However, some people argue that today’s global warming could just be part of a natural climate cycle that occurs over thousands of years” [64]. In the context of a teacher describing the controversy surrounding climate change in the media or the public, this quotation would indeed be appropriate (assuming that “some people” is taken to mean members of the public or an extreme minority of scientists).

However, one textbook in particular contains statements inconsistent with the scientific consensus, stating, “This question is at the centre of the controversy about whether humans can cause Earth’s climate to change significantly” [65]. As we have discussed above, there is no scientific question of whether humans can cause Earth’s climate to change significantly [36]–stating this in a textbook confuses the issue for students. Additionally, the word ‘may’ in the statement “Human activities … may result in climate change” [65] also suggests that there is scientific uncertainty over whether human activities are causing climate change, when no such scientific uncertainty exists. Unlike in the preceding examples, this belief is not attributed to an ambiguous outside group, but instead comes from the authoritative voice of the textbook authors themselves. The presence of such statements manufactures doubt in the minds of students where it does not exist in scientific discussions and does not serve a helpful purpose in educating scientifically literate students. Educational researchers have described best practice for discussing scientific controversies in the classroom, and exclude climate change as a possible topic both because it is not a scientific controversy (it is a social controversy) and because the scope of the debate is too broad to be handled by school students [86].

Research has shown that certain messages (such as the existence of scientific consensus) can mitigate the effect of misinformation, especially if subjects are warned of future misinformation and given advanced arguments to discredit the misinformation [87]. In some cases, it seems that the textbook writers may have been using variants of these strategies. We would suggest however that when textbooks present arguments not supported by the scientific consensus, they should be paired with refutations and that if the benefits of climate change are communicated, they should be communicated in reference to their relative importance. Phrasing the economic benefits of increased crop yield next to the economic costs of damages, for instance, would allow students to make a more reasoned comparison.

Such an approach would be more conservative than teaching strategies in subjects like health and physical education classes where normative learning objectives explicitly promote the well-being of students and society, in line with accepted social and political goals. In these subjects the minor benefits of activities like smoking (stress relief, weight loss, social acceptance in certain groups) are not always provided to students since they are dwarfed by the negative health outcomes. Certainly, one would not expect to see the statement, “Most discussions of [smoking] give the impression that the impacts of [smoking] will always be negative. However, there may also be some positive impacts” in a high school health textbook.

Finally, greater discussion of “Experts agree” and scientific consensus as a concept might benefit students, for reasons beyond climate change education. Research demonstrates that both conservatives and liberals showed poor understanding of scientific consensus, mostly taking it to mean whatever view supported their own ideological preference [88], rather than a conclusion supported by the balance of evidence in peer-reviewed literature. This is important for any issue where scientific understanding may differ from public opinion, as is the case with the anti-vaccination movement, which has caused a resurgence of easily preventable diseases in developed countries [89]. It is possible that some of these issues could be avoided with more education on the matter.

## Conclusion

We have seen that in several regards Canadian climate change education is not consistent with scientific understanding. Doubts are cast on scientific consensus in curriculum documents and textbooks, and debate is encouraged on issues that scientists have already settled. Teaching students to evaluate scientific evidence is an important skill, but it is a skill that can be nurtured by debating issues that are still under contention amongst scientists, thereby avoiding a false uncertainty over the existence of anthropogenic climate change. Curriculum documents often focus on knowledge about climate systems (“It’s climate”), missing opportunities to educate students on outcomes that would motivate them to contribute to actual solutions. Still, existing curricula and textbooks provide good models for how climate change can be communicated, and curriculum developers, unencumbered by political interference, will probably continue to improve on climate change education. Although our results are taken from the Canadian educational system, the framework that we have employed could be used to evaluate the breadth of climate change education in other jurisdictions and highlight areas for improving education to reflect the latest science while contributing to societal challenges faced by 21^st^ century citizens.

## Supporting information

S1 Text Informed consent. (DOCX)

S2 Text Interview guide. (DOCX)

S1 Table Relevant learning objectives from Canadian secondary science curricula Table 1. Learning objectives from curriculum documents were downloaded from government websites, with sources listed in the final column.
(DOCX)
