# Supplementary material for: Climate science curricula in Canadian secondary schools focus on human warming, not scientific consensus, impacts or solutions
Source: PLoS One. 2019 Jul 18;14(7):e0218305. doi: 10.1371/journal.pone.0218305 (PMC6639000; doi:10.1371/journal.pone.0218305)
Supplement: S1 Text — (DOCX) [file pone.0218305.s001.docx]

**S1 Text. Informed Consent**

This study is investigating climate change education in Canada, part of which consists of secondary science curricula in the various provinces.

Results of the study will be reported as part of a graduate degree report/presentation and may be submitted to an academic journal. Participants can withdraw from the study at any time without giving a reason or facing adverse consequences. Participants are free to refuse to answer any of the questions. Participants may also withdraw their data within two weeks of the interview.

Participants are asked to:

Participate in a digitally-recorded interview, anticipated to last one-half hour, in which you will be invited to talk about your experience in the curriculum design process, especially as it relates to the subject of climate change. Quotations reported in communications of this research will b sent to you to check for accuracy.

Recordings of the interview will be transcribed with pseudonyms. A separate pseudonym key will be created, and this pseudonym key as well as transcripts will be kept in password protected computer files. Recordings will be deleted once transcripts are finalized.

If you have any questions or concerns about the research please contact _____________ at _______________, or __________________ at ________________________

Please initial one of the following:

I consent to the placement of identifying information (my position and province, but not name) next to quotations of mine that have been approved by me for accuracy.

I wish to remain anonymous (my position and province will not be provided alongside quotations).

I understand the procedures described above. My questions have been answered to my satisfaction and I agree to participate in this study.

Signature Date
