# Supplementary material for: Climate science curricula in Canadian secondary schools focus on human warming, not scientific consensus, impacts or solutions
Source: PLoS One. 2019 Jul 18;14(7):e0218305. doi: 10.1371/journal.pone.0218305 (PMC6639000; doi:10.1371/journal.pone.0218305)
Supplement: S2 Text — (DOCX) [file pone.0218305.s002.docx]

**S2 Text. Interview Guide**

What was your official title while working on the science curriculum?

How many years of experience do you have in education?

What is the selection process for writers? (How are contributors to the curriculum chosen?)

Could you explain to me the overall process for writing the learning outcomes related to the climate change section in this course?

Were there any documents that were foundational to the curriculum writing process? (Example: “The Common Framework of Science Learning Outcomes K to 12”)

How do you decide on the amount of space given to a topic?

(Example: Ontario has an entire unit devoted to climate change whereas New Brunswick only directly addresses it in an optional unit).

(Course name) was published in (date), so the writing process was taking place roughly (years) ago. Are you aware of any updates to the curriculum regarding climate change that you would like to see, given recent developments in science?

Politically, climate change is a very controversial topic. Did that influence the approach taken to writing parts of the curriculum relating to climate change? If so, how?
