# Supplementary material for: Climate science curricula in Canadian secondary schools focus on human warming, not scientific consensus, impacts or solutions
Source: PLoS One. 2019 Jul 18;14(7):e0218305. doi: 10.1371/journal.pone.0218305 (PMC6639000; doi:10.1371/journal.pone.0218305)
Supplement: S1 Table — Learning objectives from curriculum documents were downloaded from government websites, with sources listed in the final column. (DOCX) [file pone.0218305.s003.docx]

| **Province**  **S1 Table.** Relevant learning objectives from Canadian secondary science curricula Table 1. Learning objectives from curriculum documents were downloaded from government websites, with sources listed in the final column.  far-right column. | **It’s climate** | **It’s warming** | | **It’s Us** | | | **Experts agree** | | **It’s bad** | | **We can fix it** | | **Source** |
| --- | --- | --- | --- | --- | --- | --- | --- | --- | --- | --- | --- | --- | --- |
| **Alberta** | **Science 10** |  | | **Science 10** | | |  | | **Science 10** | |  | | Education, Alberta. (2014). Science 10-20: Program of Studies. Retrieved from http://education.alberta.ca/media/654833/science10.pdf. |
| Science 10 and Science 20 are mandatory for graduation for those aiming for a university program | Unit D: Energy Flow in Global Systems: Outcomes for STS/K 1. Describe how the relationships among input solar energy, output terrestrial energy and energy flow within the biosphere affect the lives of humans and other species |  | | Unit D: Energy Flow in Global Systems: Outcomes for STS/K 4. investigate and identify human actions affecting biomes that have a potential to change climate (e.g., emission of greenhouse gases, draining of wetlands, forest fires, deforestation) and critically examine the evidence that these factors play a role in climate change (e.g., global warming, rising sea level(s)) | | |  | | Unit D: Energy Flow in Global Systems: Outcomes for STS/K 1. explain how climate affects the lives of people and other species, and explain the need to investigate climate change | |  | |  |
|  | Unit D: Energy Flow in Global Systems: Outcomes for STS/K 2. Analyze the relationships among net solar energy, global energy transfer processes—primarily radiation, convection and hydrologic cycle—and climate. |  | |  | | |  | | Unit D: Energy Flow in Global Systems: Outcomes for STS/K 3. identify the potential effects of climate change on environmentally sensitive biomes | |  | |  |
|  |  |  | |  | | |  | | Unit D: Energy Flow in Global Systems: Outcomes for STS/K 4. assess, from a variety of perspectives, the risks and benefits of human activity, and its impact on the biosphere and the climate | |  | |  |
|  | **Science 20** | **Chemistry 30** | |  | | |  | |  | |  | |  |
| **Alberta** | General Outcome 4: Students will analyze the evidence of, and assess the explanations for, natural variations in Earth’s climate over the last two million years. | C2.3s investigate the issue of greenhouse gases; identify some greenhouse gases, including methane, carbon dioxide, water and dinitrogen oxide (nitrous oxide) and analyze their contribution to climate change | |  | | |  | |  | | Education, Alberta. (2014b). Science 20-30: Program of Studies. Retrieved from http://education.alberta.ca/media/654837/sci2030_07.pdf. | |  |
| **British Columbia** | **Science Grade 10 (old)** |  | | **Science Grade 10 (old)** | | |  | | **Science Grade 10 (old)** | |  | | MOEd, BC. (2008). Science Grade 10: Integrated Resource Package 2008. Retrieved from https://www.bced.gov.bc.ca/irp/pdfs/sciences/2008sci_10.pdf. |
| Grade 10 science is mandatory for graduation | D3 describe how natural phenomena can affect climate (e.g., biosphere processes, volcanic eruptions, Coriolis effect, El Niño and La Niña) |  | | D3 describe how climate can be influenced by human activities (e.g., greenhouse gases, depletion of ozone layer) | | |  | | D3 describe how climate change affects natural systems (e.g., shrinking of the permafrost region, melting of ice shelves/caps/glaciers) | |  | |  |
|  | **Earth Science Grade 11 (new)** |  | | **Earth Science Grade 11 (new)** | | |  | | **Earth Science Grade 11 (new)** | |  | |  |
|  | Consider both natural and manmade impacts, including: - greenhouse effect – albedo – climate change |  | | Consider both natural and manmade impacts, including: - greenhouse effect – albedo – climate change | | |  | | water sources are affected by climate change:— ocean acidification — changes to ocean currents — loss of glaciers — rising sea levels | |  | |  |
|  |  |  | | **Science for Citizens Grade 11 (new)** | | |  | |  | |  | |  |
|  |  |  | | extreme weather events:  — causes and impacts  — weather and climate change | | |  | |  | |  | |  |
|  | **Environmental Science Grade 12 (new)** | **Environmental Science Grade 12 (new)** | | **Environmental Science Grade 12 (new)** | | |  | | **Environmental Science Grade 12 (new)** | | **Environmental Science Grade 12 (new)** | |  |
|  | energy balance: conduction, radiation, convection, albedo, longwave and short wave radiation, atmospheric circulation and weather | Global Warming and Climate Change: system: weather, climate, greenhouse effect, albedo effect, change over time, positive and negative feedback loops, global temperatures | | Human activities have caused changes in the global climate system. | | |  | | impacts: rising sea level, desertification, ocean acidification, polar regions, human health (e.g., changes in vectors) | | Global warming and climate change: mitigation and personal choices | |  |
| **Manitoba** | **Grade 10 Science** |  | | **Grade 10 Science** | | |  | | **Grade 10 Science** | |  | | Education, Manitoba. (2001). Senior Science 2: Specific Learning Outcomes. Winnipeg, Manitoba: Retrieved from http://www.edu.gov.mb.ca/k12/cur/science/outcomes/s2/slo.pdf. |
| Grade 10 science is mandatory for graduation | S2-4-02 Outline factors influencing the Earth’s radiation budget. |  | | S2-4-07 Investigate and evaluate evidence that climate change occurs naturally and can be influenced by human activities. | | |  | | S2-4-08 Discuss potential consequences of climate change | |  | |  |
|  | S2-4-03 Explain effects of heat transfer within the atmosphere and hydrosphere on the development and movement of wind and ocean currents. |  | |  | | |  | |  | |  | |  |
| **New Brunswick** | **Grade 10 Science** |  | |  | | |  | |  | |  | | Education, Department of. (2002). Science Grade 10. Retrieved from http://www.gnb.ca/0000/publications/curric/grade10science.pdf. |
|  | (331-3) describe examples that illustrate that the atmosphere and hydrosphere are heat sinks in the water cycle. |  | |  | | |  | |  | |  | |  |
|  | (318-1) illustrate the cycling of matter through biotic and abiotic components of an ecosystem by tracking carbon, nitrogen, and oxygen |  | |  | | |  | |  | |  | |  |
|  | **Introduction to Environmental Science 120** | **Introduction to Environmental Science 120** | |  | | |  | | **Introduction to Environmental Science 120** | |  | | DEECD. (2012). Introduction to Environmental Science 120 Curriculum. Retrieved from http://www.gnb.ca/0000/publications/curric/IntroductiontoEnvironmentalScience120Curriculum.pdf. |
|  |  |  |  |  |  |  |  |  |  |  |  | |  |
| **New Brunswick** The unit on Climate Change in the Introduction to Environmental Science 120, is one of several units that can be chosen. | Optional Unit 3: Climate Change: distinguish between the greenhouse effect, global warming and climate change. | Optional Unit 3: Climate Change: describe how Earth’s climate has changed in the short and long term, and how scientists have studied these changes. | |  | | |  | | Optional Unit 3: Climate Change: identify possible effects of climate change on NB. Include ecosystem changes, community effects, economic impact, cultural and social impacts. | |  | |  |
|  |  |  |  |  |  |  |  |  |  |  |  |  | Education, Department of(1994). Physical Geography 110: Portrait of a Planet. Fredericton, NB: Retrieved from https://www.gnb.ca/0000/publications/curric/PhysicalGeography110.pdf. |
|  | **Physical Geography 110** |  | |  | | |  | |  | |  | |  |
|  | Identify and explain the factors which control climate |  | |  | | |  | |  | |  | |  |
|  | Explain regional and chronological variations in world climate. |  | |  | | |  | |  | |  | |  |
| **Newfoundland and Labrador** |  |  | | **Environmental Science 3205** | | |  | | **Environmental Science 3205** | | **Environmental Science 3205** | | DEECD. (2010). Environmental Science 3205 Curriculum Guide (Interim Edition). St. John's, NL: Retrieved from http://www.ed.gov.nl.ca/edu/k12/curriculum/guides/science/envsci3205/ES3205_Unit_5.pdf. |
| Science 1206 is mandatory for graduation.  *This objective comes from one of three optional units that teachers can choose from in this part of the course. |  |  | | 3.60 identify environmental impacts associated with the raising of livestock and poultry. Include: i) impacts on water quality ii) reduction of biodiversity iii) impacts of climate * | | |  | | 5.17 identify that climate change can have a catastrophic affect [*sic*] on Earth. Include: (i) natural sources of greenhouse gasses [*sic*] (ii) anthropogenic sources of greenhouse gases | | 5.25 describe efforts made to address climate change. Include: (i) individual (ii) industries (iii) provincial governments (iv) federal governments (v) international agreements such as the Rio Declaration and the Kyoto Protocol. | |  |
| **Newfoundland and Labrador** |  |  | | 5.17 identify that climate change can have a catastrophic affect [*sic*] on Earth. Include: i) natural sources of greenhouse gasses (*sic*) ii) anthropogenic sources of greenhouse gases | | |  | | 5.18 describe the impacts of climate change in Canada on wildlife and natural ecosystems. Include: (i) types of vegetation (ii) shifting ecosystem boundaries (iii) biodiversity of species (iv) adaptation of species | |  | |  |
|  |  |  | |  | | |  | | 5.19 describe the impacts of climate change in forests | |  | |  |
|  |  |  | |  | | |  | | 5.20 describe the impacts of climate change in Canada on agriculture. Include: (i) length of growing season (ii) extreme weather events (iii) types of crops (iv) precipitation variability | |  | |  |
|  |  |  | |  | | |  | | 5.21 describe the impacts of climate change in Canada on fishery. Include: (i) water temperature (ii) species distribution (iii) growth rates | |  | | DEECD. (2014). Science. from http://www.ed.gov.nl.ca/edu/k12/curriculum/guides/science/#sci1206 |
|  |  |  | |  | | |  | | 5.22 describe the impacts of climate change in Canada on coastal zones (sea level changes and areas of human habitat). Include: (i) coastal erosion (ii) flooding due to expansion of ocean water caused by melting ice (iii) tectonic subsidence to (iv) Newfoundland and Labrador locations at risk | |  | |  |
|  | **Science 1206** | **Science 1206** | |  | | |  | | 5.23 describe the impacts of climate change in Canada on extreme weather events.. Include: (i) frequency (ii) intensity (iii) vulnerable areas in Newfoundland and Labrador | |  | |  |
|  | (115-6) explain how scientific knowledge evolves about changing weather patterns with new evidence about changes in ocean temperature - identify why oceans are important in weather dynamics - identify factors that are responsible for causing ocean currents | (318-1) illustrate the cycling of matter through biotic and abiotic components of an ecosystem by tracking carbon, nitrogen and oxygen - describe the processes required to cycle from carbon reservoirs to the atmosphere - describe the importance of oxygen to ecosystems - describe the significance of global warming and eutrophication | |  | | |  | | 5.24 describe the impacts of climate change in Canada on human health. Include: (i) heat stress (ii) migration of diseases | |  | |  |
| **Included especially because supporting documents make links to present day climate change for this objective. | **Earth Systems 3209** |  | |  | | |  | |  | |  | |  |
|  |  |  |  |  |  |  |  |  |  |  |  |  |  |
|  | (332-7) recognize that life forms, climate, continental positions and Earth's crust have changed over time ** |  | |  | | |  | |  | |  | |  |
| **Northwest Territories** | Uses the same science curriculum as Alberta. | | | | | | | | | | | | |
| **Nova Scotia** | **Science 10** |  | |  | | |  | |  |  | | DEECD. (2012b). Science 10: A Teaching Resource. Retrieved from http://www.ednet.ns.ca/files/curriculum/Science10TR.pdf. | |
| Science 10 is mandatory for graduation | (331-3) describe how the atmosphere and hydrosphere act as heat sinks in the water cycle |  | |  | | |  | |  |  | |  |  |
|  |  |  |  |  |  |  |  |  |  |  | |  |  |
| *Not all curriculum documents from Nova Scotia (Geology 12, Food Science) were accessible on government websites, though personal communications with educators from NS suggest that Food Science has no related objectives and Geology 12 is rarely made available to students.* | **Oceans 11** |  | |  | | |  | |  |  | |  |  |
|  | OSM-5 explain the role of the Coriolis Effect in the relationship between wind and current direction |  | |  | | |  | |  |  | |  | |
|  | OSM-6 - explain how thermohaline currents are produced |  | |  | | |  | |  |  | |  | |
| **Nunavut** | Amongst other curriculum documents, Nunavut uses Science 10, Science 20 and Chemistry 30 from Alberta | | | | | | | | | | | | |
| **Ontario** | **SNC2D** | **SNC2D** | | **SNC2D** | |  | | | **SNC2D** | **SNC2D** | | MEDU. (2008). The Ontario Curriculum Grades 9 and 10: Science. Toronto: Queen's Printer for Ontario Retrieved from http://www.edu.gov.on.ca/eng/curriculum/secondary/science910_2008.pdf. | |
| Either SNC2D or SNC2P is mandatory | D2.2 design and build a model to illustrate the natural greenhouse effect, and use the model to explain the anthropogenic greenhouse effect | D2.4 investigate a popular hypothesis on a cause-and-effect relationship having to do with climate change (e.g., the combustion of fossil fuels is responsible for rising global temperatures…) | | D2 Investigate various natural and human factors that have an impact on climate change and global warming | |  | | | D1 analyse some of the effects of climate change around the world, and assess the effectiveness of initiatives that attempt to address the issue of climate change; | D1 analyse some of the effects of climate change around the world, and assess the effectiveness of initiatives that attempt to address the issue of climate change; | |  |  |
| **Ontario** | D2.5 investigate, through laboratory inquiry or simulations, the effects of heat transfer within the hydrosphere and atmosphere | D3.8 identify and describe indicators of global climate change (e.g., changes in: glacial and polar ice, sea levels, wind patterns, global carbon budget assessments) | | D3 demonstrate an understanding of natural and human factors, including the greenhouse effect, that influence Earth’s climate and contribute to climate change. | |  | | | D1.1 analyse current and/or potential effects, both positive and negative, of climate change on human activity and natural systems (e.g., loss of habitat for Arctic mammals such as polar bears and loss of traditional lifestyles for Inuit as Arctic ice shrinks; famine as arable land is lost to desertification; an increase in water-borne disease and human resettlement as coastal lands are flooded; expansion of the growing season in some regions) | D1.2 assess, on the basis of research, the effectiveness of some current individual, regional, national, or international initiatives that address the issue of climate change (e.g., Drive Clean, ENERGY STAR, federal and provincial government rebates for retrofitting older buildings to be more energy efficient, carbon offset programs, community tree-planting programs, municipal recycling programs, Intergovernmental Panel on Climate Change [IPCC]), and propose a further course of action related to one of these initiatives | | MEDU. (2008b). The Ontario Curriculum Grades 11 and 12: Science. Toronto: Queen's Printer for Ontario Retrieved from http://www.edu.gov.on.ca/eng/curriculum/secondary/2009science11_12.pdf. | |
| **Ontario** | D2.6 investigate, through laboratory inquiry or simulations, how water in its various states influences climate patterns (e.g., water bodies moderate climate, water vapour is a greenhouse gas, ice increases the albedo of Earth’s surface) |  | | D2.3 analyse different sources of scientific data (e.g., lake cores, tree rings, fossils and preserved organisms, ice cores) for evidence of natural climate change and climate change influenced by human activity | |  | | |  |  | |  | |
|  | D2.7 investigate, through research or simulations, the influence of ocean currents on local and global heat transfer and precipitation patterns |  | | D3.3 describe the natural greenhouse effect, explain its importance for life, and distinguish it from the anthropogenic greenhouse effect | |  | | |  |  | |  | |
|  | D3.1 describe the principal components of Earth’s climate system (e.g., the sun, oceans, and atmosphere; the topography and configuration of land masses) and how the system works |  | | D3.4 identify natural phenomena (e.g., plate tectonics, uplift and weathering, solar radiance, cosmic ray cycles) and human activities (e.g., forest fires, deforestation, the burning of fossil fuels, industrial emissions) known to affect climate, and describe the role of both in Canada’s contribution to climate change | |  | | |  |  | |  | |
|  | D3.2 describe and explain heat transfer in the hydrosphere and atmosphere and its effects on air and water currents |  | | D3.5 describe the principal sources and sinks, both natural and/or anthropogenic, of greenhouse gases (e.g., carbon dioxide, methane, nitrous oxide, halocarbons, water vapour) | |  | | |  |  | |  | |
| **Ontario** | D3.6 describe how different carbon and nitrogen compounds (e.g., carbon dioxide, methane, nitrous oxide) influence the trapping of heat in the atmosphere and hydrosphere |  | | D3.7 describe, in general terms, the causes and effects of the anthropogenic greenhouse effect, the depletion of stratospheric and tropospheric ozone, and the formation of ground-level ozone and smog | |  | | |  |  | |  | |
|  | **SNC2P** | **SNC2P** | | **SNC2P** | |  | | | **SNC2P** | **SNC2P** | |  | |
|  | D3 demonstrate an understanding of various natural and human factors that contribute to climate change and global warming. | D1 analyse effects of human activity on climate change, and effects of climate change on living things and natural systems; | | D2 investigate various natural and human factors that have an impact on climate change and global warming; | |  | | | D1.1 analyse, on the basis of research, various ways in which living things and natural systems have been affected by climate change (e.g., the effect of loss of permafrost on northern roads and housing; the effect of longer growing seasons in some regions on farmers; the effect of warming oceans on coral reefs), and communicate their findings | D1.2 analyze ways in which human actions (e.g., burning fossil fuels, implementing tree-planting programs) have increased or decreased the production of greenhouse gases | |  | |
| **Ontario** | D2.2 investigate the principles of the natural greenhouse effect, using simulations, diagrams, and/or models, and compare these principles to those of an actual greenhouse | D2.4 conduct an inquiry to determine how different factors (e.g., an increase in surface temperature, an increase in water temperature) affect global warming and climate change | | D3 demonstrate an understanding of various natural and human factors that contribute to climate change and global warming. | |  | | | D2.3 use a research process to investigate a source of greenhouse gases (e.g., decaying garbage, animal digestive processes, burning biomass) and its effect on a region of Canada (e.g., melting of the polar ice cap in the Arctic, shrinking of glaciers in the Rockies) | D2.5 investigate their personal carbon footprint, using a computer simulation or numerical data (e.g. determine carbon emissions that result from their travelling to school, work, and recreation venues; from vacation travelling; from buying products imported from distant countries), and plan a course of action to reduce their footprints (e.g., a plan to increase their use of bicycles or public transit; to eat more local) | |  | |
|  | D3.1 describe the principal components of Earth’s climate system (e.g., the sun, oceans, and atmosphere; the topography and configuration of land masses) and how the system works | D3.7 identify indicators of global climate change (e.g., changes in: the mass of glacial and polar ice, sea levels, wind patterns, global carbon budget assessments, migratory patterns of birds) | | D1.2 analyse ways in which human actions (e.g., burning fossil fuels, implementing tree-planting programs) have increased or decreased the production of greenhouse gases | |  | | |  |  | |  | |
| **Ontario** | D3.2 describe the natural greenhouse effect, its importance for life, and the difference between it and the anthropogenic greenhouse effect |  | | D2.3 use a research process to investigate a source of greenhouse gases (e.g., decaying garbage, animal digestive processes, burning biomass) and its effect on a region of Canada (e.g., melting of the polar ice cap in the Arctic, shrinking of glaciers in the Rockies) | |  | | |  |  | |  | |
|  | D3.3 describe how heat is transferred and stored in both hydrospheric and atmospheric heat sinks |  | | D3.2 describe the natural greenhouse effect, its importance for life, and the difference between it and the anthropogenic greenhouse effect | |  | | |  |  | |  | |
|  | D3.4 identify different greenhouse gases (e.g., carbon dioxide, methane, water vapour, nitrous oxide), and explain how they are produced naturally in the environment |  | | D3.5 describe methods by which greenhouse gases are produced by humans (e.g., burning of biomass, chemical reactions involving pollutants) | |  | | |  |  | |  | |
|  |  |  | | D3.6 identify the natural and human causes of climate change in the world and, in particular, how Canada contributes to climate change | |  | | |  |  | |  | |
|  | **SES4U** |  | |  | |  | | | **SBI3U** |  | |  | |
|  | D1 analyse, with reference to geological records, the relationship between climate, geology, and life on Earth, and evaluate contributions to our understanding of changes in Earth systems over geological time;    **Ontario** |  | |  | |  | | | B1.2 analyse the impact that climate change might have on the diversity of living things (e.g., rising temperatures can result in habitat loss or expansion; changing rainfall levels can cause drought or flooding of habitats) |  | |  | |
|  | D1.1 analyse the relationship between climate and geology, and, using geological records, assess the impact of long-term climate change on life on Earth |  | |  | |  | | |  |  | |  | |
| **Prince Edward Island** | **Grade 10 Science 421A/431A** |  | |  | |  | | |  |  | | DEECD. (2011). Environmental Science 621A. Summerside, Prince Edward Island: Retrieved from http://www.gov.pe.ca/photos/original/eecd_ENV621A.pdf. | |
| Grade 10 Science is mandatory for graduation | (331-1) describe and explain heat transfer within the water cycle |  | |  | |  | | |  |  | |  |  |
|  | (331-3) describe how the hydrosphere and atmosphere act as heat sinks within the water cycle |  | |  | |  | | |  |  | |  |  |
|  |  |  |  |  |  |  |  |  |  |  | |  |  |
|  | **Environmental Science 621** | **Environmental Science 621** | | **Environmental Science 621** | |  | | | **Environmental Science 621** | **Environmental Science 621** | |  | |
|  | 6.2 demonstrate an understanding of how Earth's climate has changed over time | 6.6 identify the effects that climate change can have on Prince Edward Island - sea level rise - increased erosion - economic effects - social effects - species movement/loss | | 6.3 demonstrate an understanding of the greenhouse gases found in the troposphere and atmosphere - natural sources of greenhouse gases - anthropogenic sources of greenhouse gases | |  | | | 6.5 identify that climate change can have a catastrophic effect on Earth. | 6.8 demonstrate an understanding of challenges and successes made to address climate change - individual - industries - provincial governments - federal governments - international agreements  **Prince Edward Island** | | DEECD. (2005). Science 421A. Prince Edward Island: Retrieved from http://www.gov.pe.ca/photos/original/ed_sci421Aguide.pdf. | |
|  | 6.4 distinguish between the greenhouse effect and global warming |  | |  | |  | | | 6.6 identify the effects that climate change can have on Prince Edward Island - sea level rise - increased erosion - economic effects - social effects - species movement/loss |  | |  | |
| **Quebec** | **Secondary 4 Science and Technology** | **Secondary 4 Science and Technology** | | **Secondary 4 Science and Technology** | |  | | | **Secondary 4 Science and Technology** |  | |  |  |
| Either Science and Technology or Applied Science and Technology is mandatory for graduation. Quebec also has an optional program called CEGEP which is a pre-university program resulting in secondary education being a year shorter. Analysis of this program was not included in this study. | Because of their ability to absorb heat, the oceans play an essential role in regulating climate by standardizing the temperature of the Earth. | Climate change is one of the major challenges humanity will have to face. The most urgent problem is the average temperature of the Earth. | | | By burning enormous quantities of fossil fuels (coal, oil and natural gas), which generate a significant amount of CO2, and by clear-cutting forests, which hampers the natural process of CO2 transformation, we magnified the natural greenhouse effect and we are now experiencing an increase in Earth's temperature. |  | | | The permafrost is sensitive to climate change because the underground ice it contains is unstable. Warming of the permafrost can cause landslides and damage to infrastructures and alter the landscape and ecosystems. |  | | MELS. (2014). Quebec Education Program: Mathematics, Science and Technology. Retrieved February 12, 2015, from http://www1.mels.gouv.qc.ca/sections/programmeFormation/secondaire2/index_en.asp?page=math2 | |
| **Quebec** |  |  | | | Today, we are using vast quantities of nonrenewable and polluting forms of energy. This has a serious impact on the environment and especially on our climate, which raises the question of the environment's ability to adapt. |  | | | Climate change is one of the major challenges humanity will have to face. The most urgent problem is the average temperature of the Earth. |  | |  |  |
|  |  |  | | | Carbon dioxide is the most abundant greenhouse gas. Its proportion has increased over the past century because of the use of fossil fuels and the manufacture of cement. |  | | | In Québec, climate change could affect the quality of our water, endangering human health and the balance of ecosystems. It could also cause fluctuations in the level of the Great Lakes and the flow of the St. Lawrence River. These fluctuations would have various consequences for the marine transportation industry, which relies on the St. Lawrence Seaway. They would also disrupt certain ecosystems, through habitat loss or deteriorating living conditions for some species of fish. |  | |  |  |
|  | **Applied Science and Technology** |  | | | **Applied Science and Technology** |  | | | Variations in precipitation would undoubtedly affect agricultural productivity and biodiversity in Québec. Moreover, costal erosion and more frequent freezes and thaws would have an impact on the road network. |  | |  | |
|  | The carbon cycle is regulated by the interaction of continental plates, the atmosphere, the oceans and living organisms. Although plants use photosynthesis to fix carbon in nonvolatile forms, carbonate rock, precipitated or created by living beings, constitutes the largest reserve of CO2.While this gas is released during volcanic eruptions, anthropogenic emissions restore the natural balance. Certain environmental biotechnologies contribute to the chemical recycling of carbon.  **Quebec** |  | | | Certain objects, systems and products (gasoline engine, petroleum products) that meet our needs also have negative consequences. Meteorological data collected in the past fifty years show climate changes at least partly due to the use of such objects systems and products. |  | | | Finally, if permafrost thaws, soils in the far north could become unstable, affecting populations there. |  | |  | |
| **Saskatchewan** | **Science 10** | **Science 10** | | | **Science 10** |  | | | **Science 10** | **Science 10** | | Education, Ministry of. (2014a). Science 10. Retrieved from https://www.edonline.sk.ca/bbcswebdav/library/curricula/English/Science/Science_10_2014_outind_only.pdf. | |
| Science 10 is mandatory for graduation. The newer curriculum guides which were introduced for the 2014/2015 school year, were used instead of older documents. | SCI10-CD2 Investigate factors that influence Earth’s climate system, including the role of the natural greenhouse effect. | | SCI10-CD1h Research how scientists examine changes to the key indicators of climate change (e.g. CO2 concentration, global surface temperature, Arctic sea ice area, land ice mass, and sea level) to support the scientific understanding of climate change. | | SCI10-CD1 Assess the consequences of human actions on the local, regional, and global climate and the sustainability of ecosystems |  | | SCI10-CD1a Pose questions or problems relating to the effects of human actions on global climate change and the sustainability of ecosystems that arise from personal research | | SCI10-CD1i Reflect upon individual and societal behavioural and lifestyle choices that can help to minimize anthropogenic sources of climate change. | |  | |
| **Saskatchewan** | SCI10-CD2b Understand that Earth’s climate system results from the exchange of thermal energy and moisture between the sun, ice sheets, oceans, solid earth, and the biosphere over a range of timescales. | |  | | SCI10-CD1g Provide examples of human actions that have contributed to the anthropogenic greenhouse effect |  | | SCI10-CD1k Assess the current and potential future effects of ongoing changes to Earth's climate systems on the people and the environment in Saskatchewan and Canada's Arctic region | | SCI10-CD1j Develop, present, and defend a position or course of action based on personal research related to mitigating the effects of global or local climate change or to enhancing the sustainability of an ecosystem, taking into account human and environmental needs. | |  |  |
|  | SCI10-CD2c Investigate how Earth’s tilt, rotation, and revolution around the sun cause uneven heating of Earth's surface, resulting in global convection currents, the Coriolis effect, jet streams, thermohaline circulation of the oceans, and climate zones. | |  | |  |  | |  | |  | |  |  |
| **Saskatchewan** | SCI10-CD2e Explain how greenhouse gases (e.g., water vapour, carbon dioxide, methane, nitrous oxide, sulphur dioxide, and ozone), particles and clouds, and surface albedo affect the amount of solar energy absorbed and reradiated at various locations on Earth. | |  | |  |  | |  | |  | |  | |
|  | SCI10-CD2e Provide examples of positive and negative feedback mechanisms in Earth’s climate system | |  | |  |  | |  | |  | |  |  |
|  | **Environmental Science 20** | | **Environmental Science 20** | | **Environmental Science 20** | **Environmental Science 20** | | **Environmental Science 20** | | **Environmental Science 20** | |  | |
|  | ES20-TE1f Analyze the relationship between plants and climate change, including plants' roles in reducing greenhouse gases, as well as potential impacts of climate change on plant growth and distribution. | | ES20-AS2b Explore, on a variety of spatial and temporal scales, major physical, biological, and social indicators of increasing global temperatures. | | ES20-AS2c. Examine the role of policies, summits, models, and organizations, such as the Canadian Centre for Climate Modeling and Analysis (CCCma), Intergovernmental Panel on Climate Change (IPCC), and Prairie Adaptation Research Collaborative (PARC), in obtaining a high degree of consensus among scientists regarding anthropocentric climate change. | ES20-AS2c. Examine the role of policies, summits, models, and organizations, such as the Canadian Centre for Climate Modeling and Analysis (CCCma), Intergovernmental Panel on Climate Change (IPCC), and Prairie Adaptation Research Collaborative (PARC), in obtaining a high degree of consensus among scientists regarding anthropogenic climate change. | | ES20-AS2 Analyze current and potential future effects of global climate change on Earth and humans, including the need for adaptation and mitigation strategies. | | ES20-AS2 Analyze current and potential future effects of global climate change on Earth and humans, including the need for adaptation and mitigation strategies. | | Education, Ministry of. (2014b). Environmental Science 20. Retrieved from https://www.edonline.sk.ca/bbcswebdav/library/curricula/English/Science/Environmental_Science_20_2014.pdf. | |
| **Saskatchewan** |  | |  | |  |  | | ES20-AS2e. Explain the economic impact of climate change on agriculture, energy, forestry, transportation, and/or tourism in Saskatchewan. | | ES20-AS2f Examine how policy makers use scientific information, including climate model predictions, to develop adaptation and mitigation strategies to respond to the effects of climate change. | |  | |
|  |  | |  | |  |  | | ES20-AS2h. Hypothesize how life on Earth might respond to changing global climate given different scenarios change such as sea level rise, extreme weather events, water shortages, increased spread of disease, and flooding. | |  | |  | |
|  |  | |  | |  |  | | ES20-TE2d Discuss the implications of the competitive exclusion principle with respect to animals and plants in an ecosystem, including the invasive species and the potential for shifting ecozones due to climate change. | |  | |  | |
| **Yukon** | Uses the same curriculum as British Columbia | | | | | | | | | | | | |
|  |  |  |  |  |  |  |  |  |  |  |  |  |  |
